# Rapid Assessment of Octocoral Diversity and Habitat on Saba Bank, Netherlands Antilles

**DOI:** 10.1371/journal.pone.0010668

**Published:** 2010-05-21

**Authors:** Peter J. Etnoyer, Herman H. Wirshing, Juan A. Sánchez

**Affiliations:** 1 Harte Research Institute, Texas A&M University - Corpus Christi, Corpus Christi, Texas, United States of America; 2 Marine Biology and Fisheries, Rosenstiel School of Marine and Atmospheric Science, University of Miami, Miami, Florida, United States of America; 3 Laboratorio de Biología Molecular Marina (BIOMMAR), Departamento Ciencias Biológicas-Facultad de Ciencias, Universidad de los Andes, Bogotá, Colombia; University of Aberdeen, United Kingdom

## Abstract

Saba Bank is a large submerged platform (∼2200 km^2^), average depth 30 m, located 4 km southwest of Saba Island in Netherlands Antilles, Caribbean Sea. Ships traveling to and from oil terminals on nearby St. Eustatius routinely anchor on the Bank, damaging benthic megafauna. Gorgonian octocorals are vulnerable to anchor damage, and they are common and conspicuous in shallow water (15–50 m) around the banks. This prompted a rapid assessment of octocoral habitat and diversity. The primary objectives were to estimate total species richness and to characterize habitats *vis a vis* gorgonians. Landsat imagery and multibeam bathymetry were employed to identify random sites for quantitative transects. A Seabotix LBV200L remotely operated vehicle (ROV) and SCUBA were used to collect and survey to 130 m. A total of 14 scuba dives and 3 ROV dives were completed in 10 days. During that time, 48 octocoral species were collected, including two likely undescribed species in the genera *Pterogorgia* and *Lytreia*. Gorgonian richness was exceptional, but not all species were collected, because the species accumulation curve remained steeply inclined after all surveys. Two shallow-water gorgonian habitat types were identified using multidimensional scaling and hierarchical cluster analyses: 1) a high diversity, high density fore-reef environment characterized by *Eunicea* spp., *Gorgonia* spp., and *Pseudopterogorgia* spp. and 2) a low diversity, low density plateau environment characterized by *Pseudopterogorgia acerosa*, *Pterogorgia guadalupensis*, and *Gorgonia mariae*. The analyses support hypotheses of broad (∼15 km) habitat homogeneity (ANOSIM, *P*>0.05), but a significant difference between fore-reef and plateau environments (ANOSIM, *P*<0.05). However, there was some indication of habitat heterogeneity along the 15 km study section of the 50 km platform edge along the southeast rim. Our results highlight the complexity and biodiversity of the Saba Bank, and emphasize the need for more scientific exploration.

## Introduction

**Figure 1:**
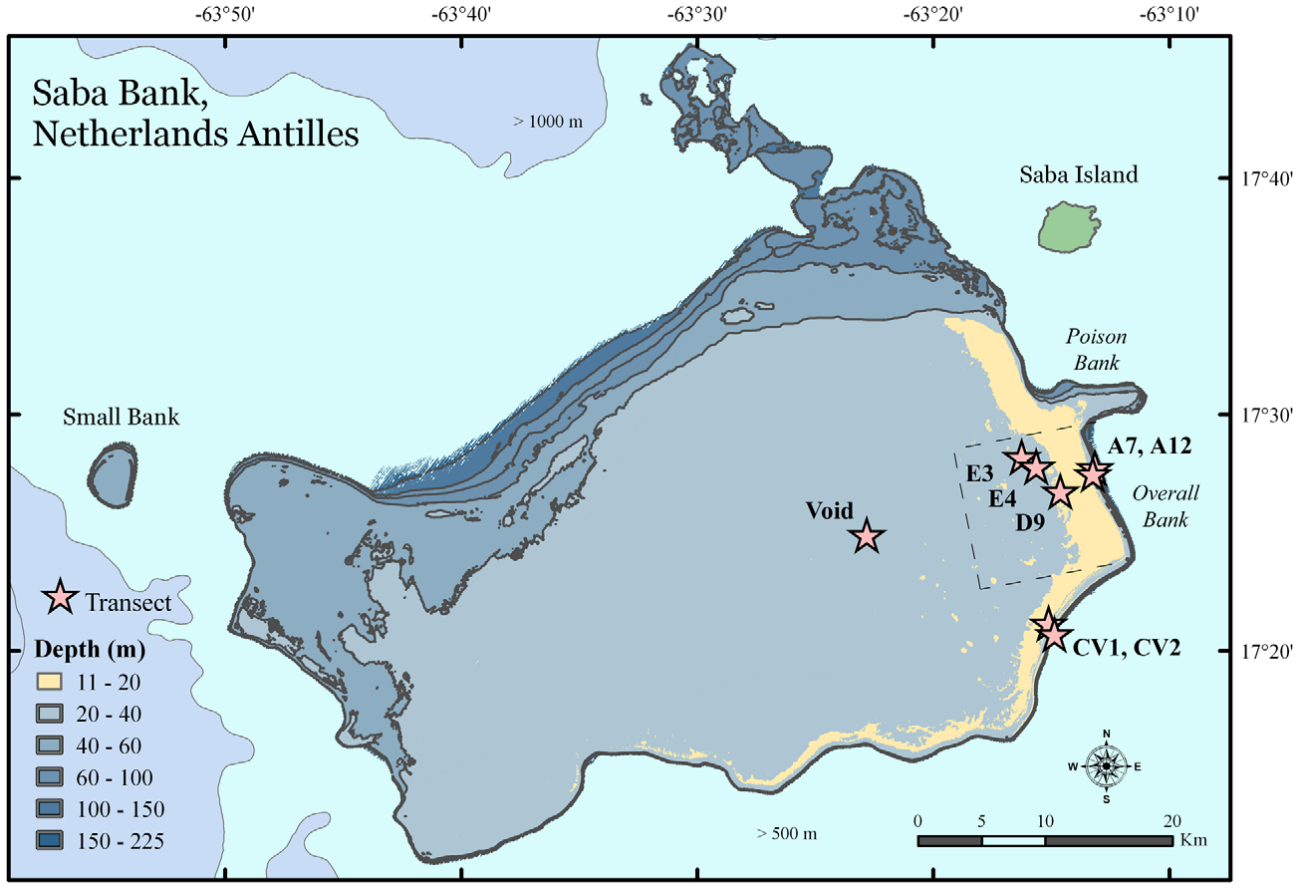
A map of Saba Bank, in the Netherlands Antilles. Saba Bank is shown relative to its nearest neighbor, Saba. A pale band of color representing 11–20 m depths represents a “fore-reef” extending 50 km along the southeast perimeter of the Bank. Red stars indicate the location of 8 quantitative transects, four on the fore-reef (A sites and CV sites) and four in the plateau (E3, E4, D9, Void) of Saba Bank's interior region. The dashed lines indicate Overall Bank, where a zonation scheme with random sites was in effect. Void was random, Conch Valley was non-random.

Saba Bank is a large submarine platform 4 km west of Saba Island across a deep-sea channel in the Netherland Antilles, Caribbean Sea. The total surface area above the 200 m isobath is ∼2200 km^2^, most of which is shallow water between 20–30 m depth, within the limits of recreational scuba diver depths. The Bank is elliptically shaped, with a 40 km short axis, and a 60 km long axis oriented ENE-WSW (Fig. 1). A linear ridge approximately 50 km in length occurs along the platform edge of the Bank. The ridge feature is raised 10–20 m, encrusted by many hard and soft tropical corals. The walls of Saba Bank are steep-sided below the platform edge, dropping off the southern rim to water deeper than 500 m between Saba Bank and the nearby island of St. Eustatius.

Geologists have traditionally referred to Saba Bank as a “remnant coastal plain” [1], an “atoll-lagoon floor, deprived of its original reef” [2], and/or a “remnant tidal marsh environment during Pleistocene or post-Pleistocene” sea level fluctuations [3]. The geological origin of Saba Bank is unclear, but Dutch SCUBA divers and surveyors [4] consider it an actively growing submerged atoll. This would make Saba Bank “the third largest atoll in the world,” though the feature never breaks surface.

**Figure 2:**
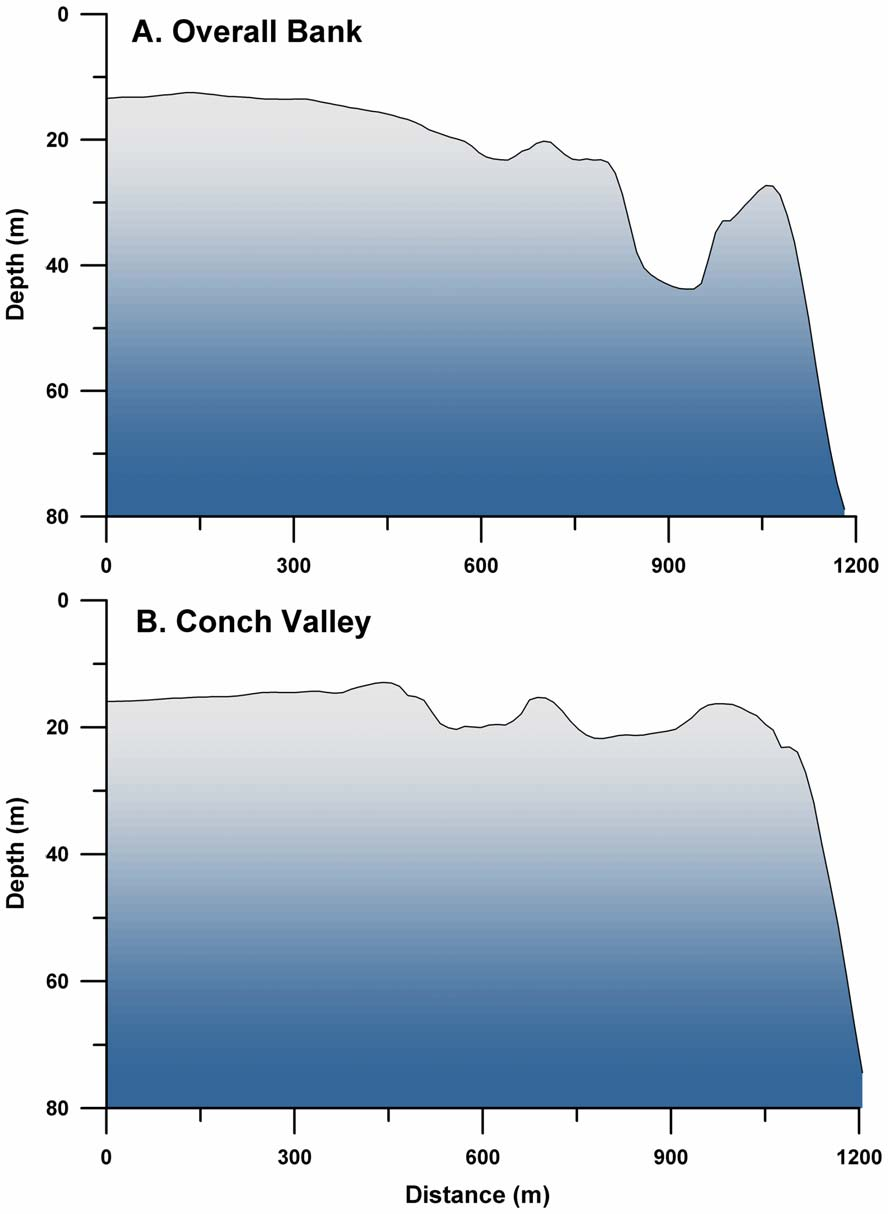
Depth profiles of the Saba Bank platform edge. Profiles of platform edge “fore-reef” dive sites on Overall Bank (A7, A12 in Figure 1) and in the Conch Valley (CV1, CV2 in Figure 1). Each fore-reef is bounded on the interior by a trough of varying depth and character. Troughs were generally sandy, with intermittent patch reefs.

Saba Bank is arguably the largest continuous shallow water habitat in the eastern Caribbean, isolated from direct terrestrial influence by a 4 km wide deep-sea channel. The platform edge is a linear ridge system, 15–30 m deep on the ESE side, characterized as sparse corals and alcyonarians, with no evidence of an interlocking coral framework [3]. This disqualified the ridge feature as a “submerged reef”, but spur and groove formations were observed during our surveys. Depth profiles of the platform edge fore-reefs indicate a series of ridges 8–10 m high, adjacent to trough-like features (Fig. 2). A classification scheme was developed to distinguish between habitat types on Overall Bank [5, Fig. 1], but this study recognizes only two classes of habitat; ‘fore-reef’ and ‘plateau’.

Saba Bank may be an important Caribbean reef habitat because it has a large extent, and it is positioned in an upstream position relative to neighboring Puerto Rico (to the northwest) and the Meso-American Barrier Reef (to the far west), in relation to the direction of flow for the Caribbean current. The Caribbean current flows east to west along the southern parts of the Antilles Ridge, turning to the northwest at higher latitudes. Long-lived invertebrate larvae (e.g. spiny lobster *Panulis argus*, Queen conch *Strombus gigas*) could theoretically disperse from Saba Bank to downstream habitats. Saba Bank is targeted for conservation because of the presence of endangered species of *Acropora* corals, and threats to habitat quality by anchor damage. The Bank is also home to small-scale local fisheries for red snapper and lobster [6], [7].

Two research expeditions studied corals on Saba Bank [4], [7]. The biological collections focused primarily on scleractinian (hard coral) species, a group with moderate richness on Saba Bank- 28 different species in 17 genera. The entire Caribbean boasts only ∼50 spp. of shallow Scleractinia [8]. Alcyonarian corals (octocorals, soft corals, gorgonians, and sea fans) are common and conspicuous on Saba Bank, but the species list for soft corals was short (14 spp., 9 genera) [4]. Gorgonian habitat types are fairly commonplace, but several groups were classified only to the genus level and these include the most diverse genera (e.g. *Pseudopterogorgia* spp., *Eunicea* spp., *Muricea* spp., *Plexaurella* spp.). Some lobster fishermen have an appreciation for the octocorals, because they believe their prey is attracted to gorgonian colonies. Gorgonians provide structural complexity for associated species of fish and invertebrates.

One intention of the scientific expedition to Saba Bank in 2007 was to improve existing knowledge of octocorals through a rapid assessment of diversity and habitat using SCUBA diving and a remotely operated vehicle (ROV). Seventeen total dives were conducted - eight dives made quantitative transects, three dives used the ROV, and six dives were dedicated entirely to sampling. The primary objectives of the expedition were (1) to document gorgonian species richness, and (2) to characterize habitat *vis a vis* gorgonians. Completeness was evaluated using a species accumulation curve with comparisons to other Western Atlantic sites [9]–[11]. Two gorgonian habitats were proposed and demonstrated, a fore-reef assemblage, and a plateau assemblage.

Our research questions asked (1) how well did we sample gorgonian diversity, (2) are the gorgonian assemblages in fore-reef and plateau environments the same or different, and, (3) is the gorgonian assemblage on Saba Bank the same or different from other sites in the Western Atlantic and wider Caribbean? The intention of the study was to encourage better conservation and management of natural resources on the Saba Bank for future generations, with particular regard to the gorgonian coral community.

## Results

Gorgonian species were common and conspicuous at all sites surveyed, except for one site, the deep-water (60 m) rubble flats south of Poison Bank, north of site A7 and A12 in Figure 1. The number of gorgonian species collected from shallow water (<40 m) was 43. The number of gorgonian species from deep water (>40 m) was 11. There was some overlap in the zones, with 6 shallow water species (*Muriceopsis flavida, Muricea laxa, Eunicea clavigera, Pseudopterogorgia (Ps.) acerosa,Ps. albatrossae*, and *Ps. bipinnata*) occurring below 40 m. A total of 48 species were collected, including two putative new species in the genera *Pterogorgia* and *Lytreia* (Table 1).

**Table 1:** List of octocoral species collected from Saba Bank.

Species	Shallow (<50 m)	Deep (>50 m)	Shallow (<50 m)	Species
*Briareum asbestinum*	X	X		Ctenocella (Ellisella) cf. elongata*
*Erythropodium* sp.	X	X		*Ctenocella (Ellisella)* sp.*
*Eunicea asperula*	X	X	X	*Eunicea clavigera*
*Eunicea calyculata*	X	X		Iciligorgia schrammi*
*Eunicea flexuosa*	X	X		*Lytreia* n. sp.*
*Eunicea fusca*	X	X	X	*Muricea laxa*
*Eunicea knighti*	X	X	X	*Muriceopsis flavida*
*Eunicea laciniata*	X	X		*Nicella* sp.*
*Eunicea laxispica*	X	X	X	*Pseudopterogorgia acerosa*
*Eunicea mammosa*	X	X	X	*Pseudopterogorgia albatrossae*
*Eunicea pinta*	X	X	X	*Pseudopterogorgia bipinnata*
*Eunicea* sp. *(tayrona)*	X			
*Eunicea succinea*	X			* = azooxanthellate
*Eunicea tourneforti*	X			
*Gorgonia mariae*	X			
*Gorgonia ventalina*	X			
*Muricea elongata*	X			
*Muricea muricata*	X			
*Muriceides* sp.	X			
*Plexaura cf. nina*	X			
*Plexaura kukenthali*	X			
*Plexaura kuna*	X			
*Plexaurella dichotoma*	X			
*Plexaurella grisea*	X			
*Plexaurella nutans*	X			
*Pseudoplexaura crucis*	X			
*Pseudoplexaura flagellosa*	X			
*Pseudoplexaura porosa*	X			
*Pseudoplexaura wagenaari*	X			
*Pseudopterogorgia americana*	X			
*Pseudopterogorgia elisabethae*	X			
*Pseudopterogorgia hummelincki*	X			
*Pseudopterogorgia rigida*	X			
*Pterogorgia cf. anceps*	X			
*Pterogorgia citrina*	X			
*Pterogorgia guadalupensis*	X			
*Pterogorgia* n. sp.	X			

The list shows species collected on Saba Bank using SCUBA for shallow (<40 m) octocorals and a remotely operated vehicle (ROV) for deep (>40 m) octocorals. The chart lists 48 species, more than three other site surveys in the greater West Atlantic. Two octocoral species are undescribed. High apparent richness is not entirely due to use of the ROV, because shallow octocorals on Saba Bank were richer than shallow octocorals in other localities.

**Figure 3:**
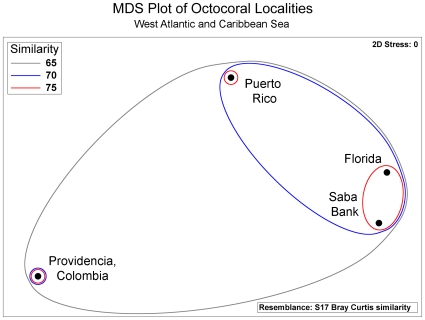
Ordination plot of Caribbean octocoral localities. A non-metric multidimensional scaling (MDS) plot based on Bray-Curtis dissimilarity measures derived from presence and absence of octocoral species in surveys of Florida [9], Colombia [10], and Puerto Rico [11].

**Figure 4:**
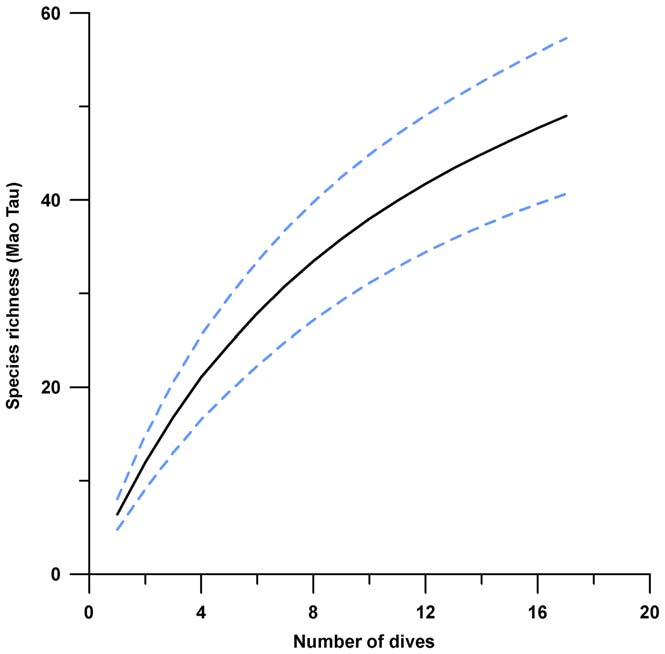
Species accumulation curve for Saba Bank gorgonians. The graph shows a sample-based rarefaction curve (in black) based on 17 shallow and deep dives. The technique employs a Mao Tau estimator [21] of expected species richness with 95% confidence intervals (in light blue). The species accumulation curve is steeply inclined, not approaching asymptote. More gorgonian species remain to be discovered on Saba Bank, though richness is already higher than other study sites in the West Atlantic.

Gorgonian richness was high, 10–30% more species than other published surveys in Colombia, Puerto Rico, and Florida [9]–[11], the assemblage was not significantly different from other western Atlantic and Caribbean sites (SIMPROF, *P*>0.05). Published species lists were >70% similar. Southeast Florida had the highest similarity (Fig. 3). The rate of species accrual on Saba Bank remained inclined after all dives (Fig. 4). Discovery rate decreased as effort increased, but the accrual curve showed no clear asymptote, so more species would be expected to occur from further sampling. Not all gorgonian species were likely to be collected, though richness was substantial.

Gorgonian diversity, richness, and abundance were significantly higher on fore-reef sites, when compared to the plateau (Mann-Whitney U- Test, *P*<0.01). The fore-reef assemblage had high species richness (S = 10–17 min/max), high diversity (Simpson mean  = 2.0), and high density of gorgonian colonies (3.5–4.7 colonies m^−2^). The plateau assemblage was lower richness (S = 3–5), lower diversity (Simpson mean  = 1.1, and lower density (0.18–1.50 colonies m^−2^) compared to the fore-reef (Table 2).

**Table 2:** Summary table of gorgonian density and diversity.

Location	Transect	Richness	Density	Diversity	Longitude	Latitude
Fore-reef	A12	17	4.188	0.846	−63.21925	17.46257
	A7	13	3.546	0.853	−63.22083	17.45819
	CV1	13	4.773	0.826	−63.25203	17.35279
	CV2	10	4.637	0.753	−63.24800	17.34500
Plateau	D9	5	1.500	0.628	−63.24387	17.44595
	E4	5	0.455	0.680	−63.26070	17.46343
	E3	4	0.182	0.750	−63.27110	17.47018
	Void	2	0.182	0.375	−63.38047	17.41512

Transects in Saba Bank's fore-reef environment (A12 - CV2) had significantly more gorgonians, and higher diversity (Mann-Whitney U Test, *P*<0.01) than transects in the plateau environment (D9 - Void). Richness is the number of octocoral species present. Density values are in colonies m^−2^. The diversity measure is Simpson's index (in the natural log form).

**Figure 5:**
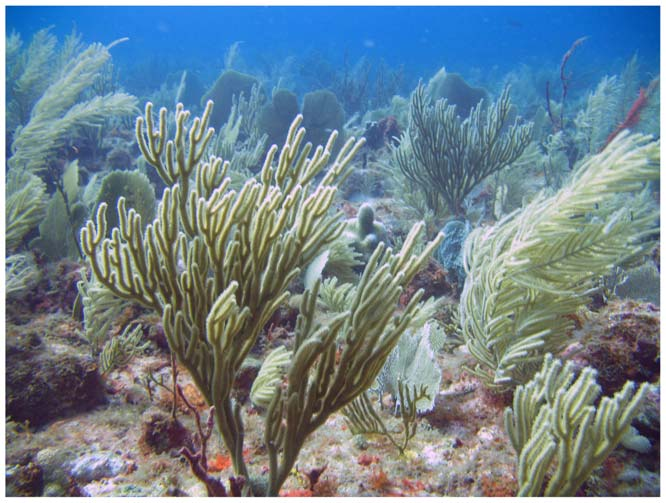
High-diversity gorgonian assemblage on the Saba Bank fore-reef. An image from one of the “A” sites on the fore-reef of Saba Bank (∼25 m) shows an assemblage characterized by sea fans (*Gorgonia ventalina*), sea plumes (*Pseudopterogorgia americana*), and the *Eunicea/Plexaura* sp. complex, *Eunicea fleuxosa*, center. The fore-reef environment consists of a series of reef crests near the platform edge on Saba Bank [4] with diverse and abundant octocoral species.

**Figure 6:**
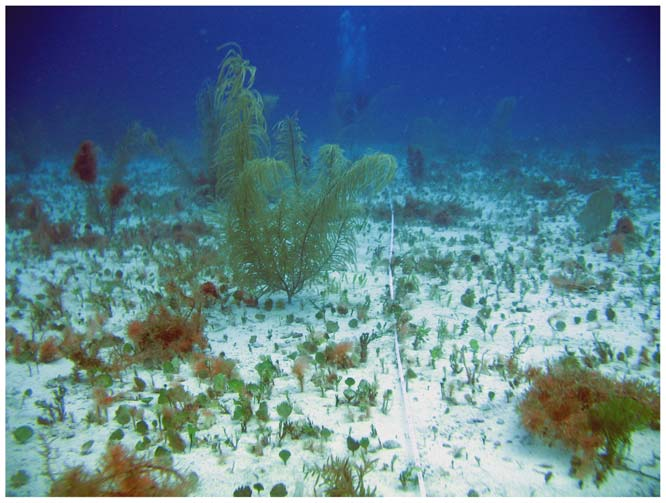
Low-diversity gorgonian assemblage on the Saba Bank plateau. An image from one of the “E” sites in the Saba Bank plateau region shows a large sea plume (∼1.5 m- *Pseudopterogorgia acerosa*) and scattered sea fans *Gorgonia mariae* attached to a hard carbonate platform covered with a thin veneer of sediment. The plateau region is distinct from the fore-reef, consisting of reef flat and lagoon bottom environments [4].

The *Eunicea* species complex, *Gorgonia* spp. sea fans, and *Pseudopterogorgia* sea plumes were typical in the fore-reef environment (Fig. 5a). Large, sparsely spaced *Ps. acerosa* and *Pterogorgia (Pt.) guadalupensis* sea plumes and small *G. mariae* sea fans comprised the plateau assemblage (Fig. 6).

Two octocoral species were collected that may be new to science. *Lytreia* n. sp. was collected twice from 50 m depth near the A zone. *Pterogorgia* n. sp. was collected twice from 20 m depth in the D zone. Specimens were observed with other *Pterogorgia* species in the same habitat, side-by-side with species exhibiting different colonial morphologies. One species previously reported [4] was not encountered, *Gorgonia flabellum*. *G. flabellum* is generally considered endemic to the Bahamas or very shallow fore-reef environments [JAS, *pers. obs*.].

**Figure 7:**
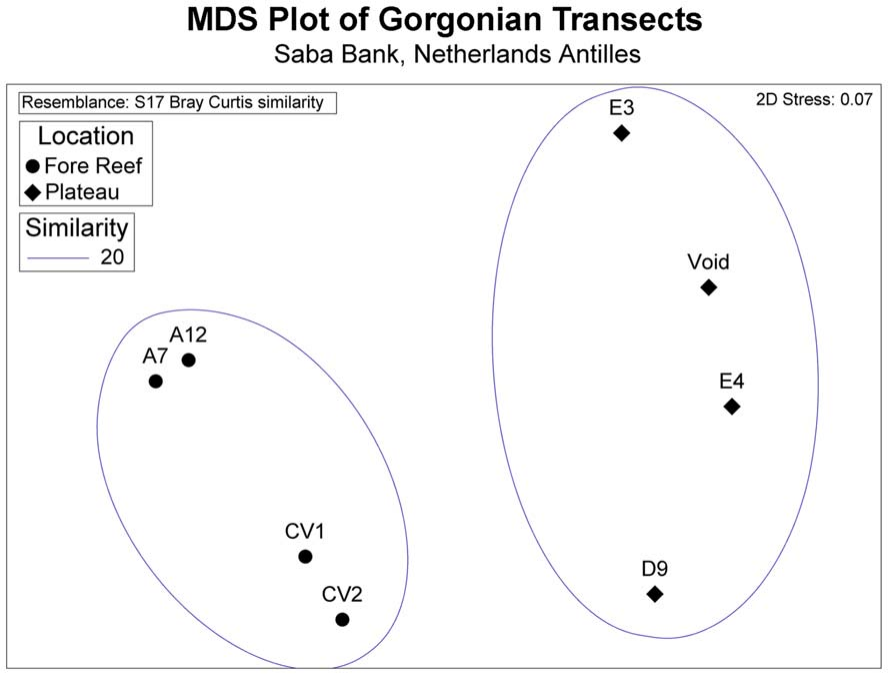
Ordination plot of Saba Bank transects. A non-metric multidimensional scaling (MDS) plot based on Bray-Curtis dissimilarity measures derived from species abundance values in 8 quantitative transects. Fore-reef sites (A, CV) are significantly different (ANOSIM, *P*<0.05) from plateau sites (D, E, and Void), but not significantly different from each other. There is good separation between fore-reef and plateau sites, with slight prospect of a misleading representation (2D stress <0.10).

Fore-reef assemblages were significantly different from plateau assemblages (Fig. 7 - ANOSIM, *P*<0.05), but not significantly different from each other (ANOSIM, *P*>0.05) using raw, untransformed data. Root transformed data gave the same results. Transects within the same zones (e.g. A zone transects A7, A12) were more similar to each other than to sites in other zones (e.g. Conch Valley transects CV1, CV2). Transects in the plateau (E4, E7) were not significantly different from sites D9 and the “Void” (ANOSIM, *P*>0.05), though these sites were 15 km distant.

The MDS ordination supported two groups less than 20% similar. Stress values in the MDS were low (Fig. 7, stress  = 0.07), corresponding to a good ordination with slight prospect of a misleading representation [12]. These results support the hypothesis of two different gorgonian assemblages on Saba Bank – a diverse and abundant assemblage on the fore-reef, and a low diversity, low-density assemblage on the plateau.

Plateau transects (D9, E3, E4, Void) with low richness and abundance were at the horizontal opposite extreme of fore-reef transects (A7, A12, CV1, CV2) with high richness and abundance in the MDS plot (Fig. 7). The axis also represents a gradient in *Eunicea* spp. abundance, descending from left to right. *Eunicea* spp. were uncommon in the plateau. Fore-reef sites with highest richness (A7, 17 spp.) were vertically opposite fore-reef sites with lowest richness (CV2, 10 spp.) in the MDS plot, and plateau sites with high abundance (D9) were opposite sites with lowest abundance (Fig. 7). This vertical axis could also represent a gradient in abundance of shared species. *Ps. acerosa* and *Pt. guadalupensis* (at CV1 and D9) would pull in the downward direction with *Plexaurella dichotoma* (at E4 and A12) pulling in the upward direction. To summarize for brevity, gradients in richness and abundance contributed to the differences between sites, but rare and cosmopolitan species were suspected to be important interactors. Rare species were defined as species that occurred in only one site. Cosmopolitan species were defined as species that occurred in more than one site.

Four cosmopolitan species occurring at more than 4 sites accounted for 50% of the difference between habitats: (*Gorgonia mariae, Pseudopterogorgia acerosa, Eunicea fleuxosa*, and *Ps. americana*). *E. fleuxosa*, *Ps. acerosa, and Ps. americana* explained the community patterns best. No single species dominated the comparison, because 13 species were necessary to account for 90% of the difference. The pattern that emerged from the inverse analysis was a central core of common species, with radiations outward from this center, in 4–5 directions. The periphery of the MDS plot was occupied by rare species that occurred once (e.g. *Pterogorgia* n. sp. [D9], *Muricea muricata* [E4], *Pseudopterogorgia elisabethae* [A12]). The distinction between rare and cosmopolitan species was consistent with this interpretation. Stress was moderate (0.13), suggesting the two dimensional plot was a satisfactory representation of Bray-Curtis distance.

To evaluate the importance of taxonomic resolution in the habitat classification, abundance values for congeners were summed to produce an average density for each transect by genus. *Eunicea* (n = 11), *Pseudopterogorgia* (n = 7), and *Pterogorgia* (n = 4) were the most speciose genera. Generic assemblages on the fore-reef and plateau were significantly different, consistent with the species-level diagnosis (ANOSIM, *P*<0.05).

Transects in both habitat types were separated by 15 km, but the clusters indicate some degree of homogeneity within habitats, because sites 15 km away were more similar than adjacent sites in different habitats. Fore-reef transects clustered more closely together than plateau sites, suggesting fore-reef transects were more similar to each other than plateau transects. We found strong evidence for two habitat types, with good evidence for broad homogeneity within zones, but only subtle evidence of heterogeneity between remote stations.

We reject the null hypothesis of no difference between fore-reef and plateau environments with good confidence, based on multivariate (ANOSIM, *P*<0.05) and univariate techniques (Mann-Whitney U test, *P*<0.01) with robust distinctions across methods. Further exploration of Saba Bank warranted, based on the continuous climb of the species accrual curve (Fig. 4), the discovery of two potentially new species, and good evidence of habitat homogeneity (and heterogeneity) along a biologically rich 50 linear km fore-reef feature.

## Discussion

The October 2007 survey results added 28 species and 7 genera to the list of gorgonian octocorals known to occur on Saba Bank. Surveys resulted in a four-fold increase in knowledge of gorgonian species richness. Richness was higher than other sites in the region, but the species composition was not significantly different from other Western Atlantic [8], [10] and Caribbean [9] sites. The use of the Seabotix ROV did not account entirely for the difference in species recovery between Saba Bank and other localities, because we collected more shallow octocorals, as well as more species overall.

Our data indicate we cannot assume we collected all of even the *most common* species. Most species are rare, so an asymptotic function implies only that the most common species were collected. Our species accrual curve did not approach asymptote, so both rare and common species remain to be discovered. This is remarkable, considering the shallow gorgonian assemblage is already rich when compared to other places. Saba Bank has at least 43 zooxanthellate octocoral species, compared to Florida (S = 39), Providencia Island, Colombia (S = 32), and Puerto Rico (S = 38). It would be useful to compare species accrual curves at each of these localities.

There is good potential for a diverse assemblage of deep-water gorgonians on Saba Bank. Conditions appear to be favorable to octocorals, because richness is high and different habitat types occur. Conditions on Saba Bank support a broad variety of octocorals over large areas, so it is logical to assume these conditions would prevail in deep-water, too. This study collected 11 deep-water species in 3 dives. More deep-water exploration of Saba Bank's is necessary, particularly in light of the presumed habitat association for Caribbean spiny lobster, which frequent deep-water.

The likely new species of *Lytreia* was exciting because the organism was recovered using the ROV to 130 m depth. The diagnostic characters of the organism were characteristic of *Lytreia* but different from the one species in the genus, *L. plana*. *Lytreia* is presently monophyletic [13]. The likely new species of *Pterogorgia* was unanticipated because the discovery occurred in shallower, more accessible water depths on the plateau in the “D zone”. New species in two depth zones suggest further explorations of the deep southern slope and the interior region would be likely to yield new discoveries.

The morphological novelty of the new *Pterogorgia* sp. was conspicuous. The genus is presently comprised of three species, *P. guadalupensis, P. citrina*, and *P. anceps*; characterized by colonies with branches that are flattened or triangular in cross section. Polyps retract into calyces that form a common groove (*P. anceps* and *P. guadalupensis*) or into close-set individual calyces (*P. citrina*) [13]. Saba's *Pterogorgia* sp. had flattened blades characteristic of *P. gudalupensis*, but calyces typical of *P. citrina*. Microscopic observation of the diagnostic calcareous sclerites identified differences between Saba Bank *Pterogorgia* sp. and its likely sister species. A morphological and molecular description of the new species will be reported elsewhere. Interestingly, all three *Pterogorgia* spp. were collected sympatrically from the discovery site (D9).

Two large (30 cm) and healthy bright red *Nicella* sea fans were collected from 60 m depth with brittlestars attached. *Nicella* occurs 300–400 m deep throughout the Caribbean. The living *Nicella* colonies had bright white retractile polyps that lent a pattern to the living colonies, so it was interesting to see the color and pattern faithfully reproduced on the brittlestars. Visual camouflage was expected in the photic zone, but less expected in the mesophotic. The photic zone likely extended to depths of 100 m or more on Saba bank, because visibility was generally very good most days. Naturally, the observations were biased by the fact that dives were conducted only when the sea state would accommodate the long trip to Saba Bank.

The idea of two distinct Saba Bank gorgonian habitats was supported by multivariate analysis techniques (Fig. 7). The gorgonian assemblage on the fore-reef was particularly rich, up to 17 spp. occurred in one transect. The assemblages were characterized by *Eunicea* spp., *Gorgonia* spp., *Plexaura* spp., *Pseudoplexaura* spp., and *Pseudopterogorgia* spp. in relatively high densities (Fig. 5). The explanation for the richness of fore-reef assemblages was that the substrate is high relief habitat (∼10 m) adjacent to a steep precipice bathed by impinging currents. There was good potential for localized upwelling, which could benefit octocorals. Alcyonarians covered >85% of substrate in some places, many hundreds of colonies were evident.

Plateau environments were fundamentally different. The gorgonian assemblage on the plateau was relatively depauperate, characterized by *Pseudopterogorgia* spp., *Gorgonia* spp., and *Pterogorgia* spp. in low densities (Fig. 6). The average sea plume colony on the plateau was probably larger than gorgonian colonies on the fore-reef. Sea plumes reached >2.5 m height above the surrounding pavement. The current flow was characterized by persistent surge. The assemblage was low density, but biomass and abundance was substantial, because colonies were large, but sparsely spaced. The habitat is potentially vast, and may occur throughout a large part of Saba Bank's interior.

Gorgonian habitats were a mosaic of endemism, richness, and abundance superimposed over a background of broad scale (15 km) homogeneity. Conch Valley was the southernmost fore-reef site we surveyed, not significantly different from sites in the A zone, 15 km to the northeast, but the fore-reef zones were separated in the ordination space, so differences were present. Conch Valley's fore-reef had high abundance of gorgonians, but richness was likely underestimated, only two transects were performed.

The normal analysis of the ordination helped to illustrate that species richness and colony density were important discriminators between sites, but the inverse analysis showed rare species were also important to consider. We found five rare species in 8 sites (18% of species were rare). These five species “typified” the sites where they occurred. In a sense, rare species marked a site's departure from some background level of abundance in a small group of 4–5 cosmopolitan species. The utility of this message is that 28 gorgonian species could be classified between two types, rare and cosmopolitan. Both types were important.

Beta diversity, i.e., number of different assemblages at a local scale, is known to occur along a continuum of environmental conditions [14]. Depth, wave exposure, hard substrate availability, and periodic disturbance are the structuring forces in the octocoral community [see 15–17]. The other pattern structuring gorgonian assemblages is the availability of plankton resources for heterotrophic octocorals, i.e., species that lack zooxanthellae. Azooxanthellate species are distributed at the edges of reefs where the resource is available, and not yet been depleted by suspension feeders [10], [18]–[19].

In general, gorgonian populations on Saba Bank appeared healthy, with only a few instances of fungal infections of *Aspergillosis* on *Gorgonia ventalina*. Species associations were rare, or inconspicuous, other than brittlestars on *Nicella*, and a few basket stars wrapped around *Pseudopterogorgia* axes. Hard corals on the fore-reef area near Poison Bank appeared to be degraded. Many hard coral colonies were bleached and dead, overgrown by calcareous algae. There is precedent for an inverse relationship between hard coral cover and soft coral cover [20].

Our studies were limited to the southeast corner of the Saba Bank, but the results offer some insights on the probable character of gorgonian habitats throughout the Bank. The high diversity/high abundance habitat is the fore-reef (i.e. the “A zone”) of fringing reef habitat along 50 km of the Saba Bank perimeter. A lower diversity plateau habitat underlain by hard carbonate pavement appears to dominate the interior parts of the Bank. The latter would be expected to occupy a larger part of the Bank.

Octocoral colonies were vulnerable to damage. Large toppled gorgonian colonies were observed in the sand channel approaching Conch Valley. The area is heavily fished by lobstermen using pots and traps. No similar damage was observed at other sites. This manuscript is part of a larger effort to assess biodiversity of the benthic habitat and to mitigate anchor damage on Saba Bank through the establishment of a Particularly Sensitive Sea Area. Oil tankers en route to and from St. Eustatius regularly drop anchor on the bank, damaging benthic megafauna.

Based on our findings, scenarios for a zoning scheme to mitigate damage to octocorals would range from: 1) the status quo, no control, to 2) the restriction of tanker anchors from the fore-reef perimeter, or 3) the restriction of tanker anchors to a designated sandy zone in the Bank interior, if one exists, or 4) a complete ban prohibiting anchor damage on the bank. The status quo is likely to impact octocorals adversely, because they are broadly distributed and potentially long-lived. Colonies are vulnerable to overturning. The second option would protect the most diverse assemblages of octocorals. The third option would impact low diversity habitat, but habitat loss in a small area could be mitigated by habitat protection in other parts of the bank. The fourth option, a complete anchor ban, would reduce impacts to all habitats, but octocoral colonies would still be vulnerable to bottom-contact fishing gear.

Recommendations for further study include: 1) expansion of the study area to include more fore-reef sites along the southeast rim of the Bank; 2) increased exploration of the heretofore unknown 500 m vertical escarpment in the southern parts, and deep raised topographies in the northern parts; 3) the incorporation of isolated features (e.g. mounds and ridges) into the research design to test the hypothesis of habitat homogeneity in the plateau; and 4) the continued pursuit of greater sample sizes in all zones (especially B and C zones) to confirm these preliminary findings. It would also be interesting to test lobstermen's claims of affinity between octocorals and spiny lobsters, because both these animals are likely to occur in deep, unexplored parts of Saba Bank.

## Methods

One of the primary objectives was to collect and identify as many gorgonian species as possible using oxygen enriched air (nitrox) with scuba and a Seabotix LBV200L remotely operated vehicle (ROV) rated to 200 m depth on separate boats. The scuba boat was a 28 ft. vessel powered by twin 115 HP engines. The ROV boat a 35 ft. lobster fishing boat equipped with a winch for hauling traps from deep water. A total of 14 scuba dives and 3 ROV dives were completed over the course of the 10-day expedition, October 19–31, 2007. The first four scuba dives were dedicated to primarily specimen collection and photography. Two additional opportunistic dives on random sites collected specimens not found on other sites. Eight other dives were used to conduct quantitative transects in two different “habitat zones”, the fore-reef and the plateau.

All gorgonian specimens were photographed *in situ* before 10–15 cm branch segments were cut from the distal tips of branches, and preserved in 95% ethanol. These were identified to species using microscope slide preparations of sclerite morphology, using established octocoral protocols [13]. Permission for sample collection was granted through the Saba Conservation Foundation. Following identification and analysis, preserved specimens were curated at the Department of Invertebrate Zoology in the Smithsonian Institution National Museum of Natural History (NMNH). Records were uploaded to the Smithsonian NMNH database with in-situ images.

The research was primarily concerned with the southeastern quadrant of Saba Bank. A block design was established for “Overall Bank” south of Poison Bank, but survey sites in Conch Valley and on the plateau were haphazard. Within Overall Bank, sites were random, zones were labeled A–E, with A representing the “fore-reef” environment and E representing the “lagoon environment”. Zonation was simplified to two zones for this study – the fore-reef (A sites) and the plateau (D, E sites).

A total of 8 quantitative dive transects were conducted in five different zones. Four transects (A7, A12, CV1, CV2) were assigned to the fore-reef, and four other transects (D9, E3, E4, Void) were assigned to the plateau. The Void site was given a unique name, as it is ∼15 km from the other plateau sites. Fore-reef transects were random at two sites in the A zone (A7 and A12), and haphazard on the southeast rim called Conch Valley 1 (CV1) and Conch Valley 2 (CV2). Fore-reef sites CV1 and CV2 are ∼15 km from the A zone fore-reef sites (Fig. 1). Plateau sites appeared to be clustered on Overall Bank, but sites were random. One transect occurred in the D zone at D9, two in the E zone at E3 and E4, and one in the unmapped region of the Bank interior referred to as “Void”. No gorgonian transects were conducted in zones B and C.

Quantitative transects were conducted by 2 divers working in quadrats along a 50 m transect tape. Octocoral colonies were enumerated by species in 1 m square quadrats placed every 5 meters on either side of the transect tape (11 quadrats per diver, 22 total quadrats per transect). The numbers of colonies in each quadrat were summed together, pooled from both divers, then divided by 22 m^2^ to achieve species density values in units of colonies m^−2^. The resulting data matrix listed species density values for 28 species (variables) along eight transects (observations). This is not be confused with the total number of gorgonian species collected on Saba Bank (48 spp.), because species were collected independently of the transects.

Univariate techniques used the Mann-Whitney U Test and Simpson's diversity index in the form (1 - λ). Multivariate ordination techniques were used to plot similarity, and to test the null hypothesis of no difference between (a) habitats at the species level, (b) habitats at the genus level, and (c) localities at the species level. The hypothesis of no difference between localities compared the Saba Bank species list to other West Atlantic gorgonian lists from Florida [9], Colombia [10], and Puerto Rico [11]. No *a priori* difference was assumed between western Atlantic and Caribbean localities. Sample-based rarefaction (species accumulation) curves were plotted with 95% confidence intervals using a moment based indicator of expected species richness called Mao Tau in EstimateS 8.0 software [21]. The input matrix used abundance or incidence values, depending on the dive type.

Three matrices were employed. The first was prevalence data with species as variables, and sites as observations. The second was prevalence data with genera as variables. The third matrix was incidence data, with species as variables and localities as observations. A resemblance matrix was produced for each matrix using the Bray-Curtis distance measure on raw, untransformed data in PRIMER 6.1 software [12]. The resemblance matrix informed non-metric multidimensional scaling (MDS) plots and group averaged hierarchical dendrograms of sites, species, and localities.

Hypotheses of no difference between sites were tested using ANOSIM Global-*R* and the SIMPROF statistics in PRIMER 6.1. ANOSIM was used for *a priori* comparisons between sites and SIMPROF for *a posteriori* comparisons between localities. ANOSIM Global *R*-values are compared to a null distribution of rank distances generated by the random rearrangement of factors (fore-reef, plateau) and site labels (A7, A12, CV1, CV2, D9, E4, E7, Void) in order to estimate the probability of the same ordination, or Bray-Curtis distance configuration, occurring as a result of random chance. Similarity Percentage (SIMPER) analysis, BEST analysis, and inverse analysis were used in PRIMER 6.1 to understand which species were driving differences between sites.
